# Determinants of hyperglucagonemia in pediatric non-alcoholic fatty liver disease

**DOI:** 10.3389/fendo.2022.1004128

**Published:** 2022-09-05

**Authors:** Katharina Maruszczak, Konrad Radzikowski, Sebastian Schütz, Harald Mangge, Peter Bergsten, Anders Forslund, Hannes Manell, Thomas Pixner, Håkan Ahlström, Joel Kullberg, Katharina Mörwald, Daniel Weghuber

**Affiliations:** ^1^ Department of Pediatrics, Obesity Research Unit, University Hospital Salzburg, Paracelsus Medical University, Salzburg, Austria; ^2^ Department of Mathematics, Paris Lodron University, Salzburg, Austria; ^3^ Clinical Institute of Medical and Chemical Laboratory Diagnostics, Medical University of Graz, Graz, Austria; ^4^ Department of Medical Cell Biology, Uppsala University, Uppsala, Sweden; ^5^ 5Department of Women’s and Children’s Health, Uppsala University, Uppsala, Sweden; ^6^ Department of Pediatric and Adolescent Medicine, Salzkammergutklinikum Voecklabruck, Voecklabruck, Austria; ^7^ Department of Radiology, Uppsala University, Uppsala, Sweden & Antaros Medical, BioVenture Hub, Mölndal, Sweden

**Keywords:** glucagon, childhood obesity, NAFLD, pediatrics, hyperglucagonemia

## Abstract

**Objective:**

Over the years, non-alcoholic fatty liver (NAFLD) disease has progressed to become the most frequent chronic liver disease in children and adolescents. The full pathology is not yet known, but disease progression leads to cirrhosis and hepatocellular carcinoma. Risk factors included hypercaloric diet, obesity, insulin resistance and genetics. Hyperglucagonemia appears to be a pathophysiological consequence of hepatic steatosis, thus, the hypothesis of the study is that hepatic fat accumulation leads to increased insulin resistance and impaired glucagon metabolism leading to hyperglucagonemia in pediatric NAFLD.

**Methods:**

132 children and adolescents between 10 and 18 years, with varying degrees of obesity, were included in the study. Using Magnetic Resonance Imaging (MRI) average liver fat was determined, and patients were stratified as NAFLD (>5% liver fat content) and non-NAFLD (<5%). All patients underwent a standardized oral glucose tolerance test (OGTT). Additionally, anthropometric parameters (height, weight, BMI, waist circumference, hip circumference) such as lab data including lipid profile (triglycerides, HDL, LDL), liver function parameters (ALT, AST), uric acid, glucose metabolism (fasting insulin and glucagon, HbA1c, glucose 120 min) and indices evaluating insulin resistance (HIRI, SPISE, HOMA-IR, WBISI) were measured.

**Results:**

Children and adolescents with NAFLD had significantly higher fasting glucagon values compared to the non-NAFLD cohort (p=0.0079). In the NAFLD cohort univariate analysis of fasting glucagon was associated with BMI-SDS (p<0.01), visceral adipose tissue volume (VAT) (p<0.001), average liver fat content (p<0.001), fasting insulin concentration (p<0.001), triglycerides (p<0.001) and HDL (p=0.034). This correlation equally applied to all insulin indices HOMA-IR, WBISI, HIRI (all p<0.001) and SPISE (p<0.002). Multivariate analysis (R² adjusted 0.509) for the same subgroup identified HIRI (p=0.003) and VAT volume (p=0.017) as the best predictors for hyperglucagonemia. Average liver fat content is predictive in pediatric overweight and obesity but not NAFLD.

**Conclusions:**

Children and adolescents with NAFLD have significantly higher fasting plasma glucagon values, which were best predicted by hepatic insulin resistance and visceral adipose tissue, but not average liver fat content.

## Introduction

Non-alcoholic fatty liver (NAFLD) is associated with obesity, insulin resistance and type 2 diabetes. NAFLD has progressed to become the most frequent chronic liver disease in children and adolescents (1). The estimated prevalence of NAFLD in children is 12.5% (95% CI: 9.2% to 16.7%) in youth with overweight, 36.1% (95% CI: 24.6% to 49.9%) with obesity and 2.3% (95% CI: 1.5% to 3.6%) in children with normal weight, boys thereby exhibiting a higher prevalence than girls (2). NAFLD in adolescents has recently been shown to substantially increase the risk of T2DM in children in general, with as many as one in three children with NAFLD having abnormal glucose metabolism (3–5). Obesity and insulin resistance are known to add up to this development, but beta cell and alpha cell function, respectively, have been shown to alter insulin secretion and cause a hyperglucagonemic state in adults (6–9). The pancreatic hormone glucagon is a key player in blood glucose regulation and the pathophysiology of diabetes (10). Increased fasting levels of glucagon can be found in T2DM patients and in subjects with obesity and normal glucose tolerance (11, 12). Hypoglycemia is one of the major stimulants for glucagon secretion triggering hepatic glucose production (13). NAFLD consequently leads to impaired hepatic glucagon signaling resulting in hyperglucagonemia (13). The concept of the liver-alpha cell axis provides a potential causal explanation for this phenomenon (14, 15). Previously, we demonstrated that hyperglucagonemia is associated with hyperinsulinemia, high plasma free fatty acids (FFAs), high plasma triglycerides, visceral adiposity, and impaired glucose tolerance as early as during childhood (3). To date, there is a lack of studies assessing alpha cell function in pediatric NAFLD. In this study we hypothesize, that hepatic fat accumulation leads to increased insulin resistance and impaired glucagon metabolism followed by hyperglucagonemia in pediatric NAFLD.

## Material and methods

### Study population and design

The ethics approval for the study was obtained from the ethical committee of Salzburg (Number: 1544/2012) and the Uppsala regional ethical review committee (number 2012/318). The study was carried out according to the Declaration of Helsinki. Written informed consent was achieved from all participants and at least one of their caregivers.

A cross-sectional retrospective study was conducted in two study centers, Uppsala University Hospital, Sweden, and at Paracelsus Medical University Hospital in Salzburg, Austria. Data analysis was based on the material obtained by the BETA JUDO study (BETA cell function in Juvenile Diabetes and Obesity, FP7-HEALTH-2011-two-stage, project number: 279153). In total, 206 patients received MRI scans for body fat composition assessment. The MRI scans (liver fat content, body fat composition, abdominal visceral and subcutaneous fat) as previously described (16) was determined by 1.5 T clinical MRI systems from Philips Medical System (Netherlands). Patients aged 10-18 years with overweight or obesity according to the WHO criteria (BMI-SDS>1.26) and control subjects without overweight or obesity were included. Exclusion criteria were presence of chronic liver disease, known pre- and diabetes, psychiatric disorders, allergies, alcohol intake, consuming steatogenic drugs, endocrine disorders and/or hereditary causes of liver disease. 184 patients (target group) fulfilled the overweight and obesity selection criteria, and 22 patients completed the control group. The target group was divided further into a NAFLD and non-NAFLD (defined by liver fat content ≥5 and ≤ 5 %) group. After matching for BMI-SDS a sub-sample of 132 patients was selected.

### Anthropometric and blood pressure measurements

Height and weight were assessed by standardized and calibrated scales (Seca, Hamburg, Germany) and stadiometers (Uppsala: Ulmer (Busse Design + Engineering GmbH; Elchingen, Germany); Salzburg: Seca). The BMI-SDS was calculated with Microsoft Excel add-in LMS Growth using WHO growth report (17). Waist circumference (cm) was measured midway between the superior border of the iliac crest and lowest rib. Systemic blood pressure was measured using a standardized clinical aneroid sphygmomanometer (Uppsala: CAS 740; CAS Medical Systems, Inc, Branford, Conn; Salzburg: Carescape V100; Dinamap Technology/GE, Vienna, Austria), two measurements were taken, and the means were used for analyses. According to Tanner, patients were categorized into their puberty stages, prepubertal (group 1 = Tanner I), pubertal (group 2 = Tanner II–IV), and postpubertal (group 3 = Tanner V).

### Blood sampling and biochemical measurements

All blood sample parameters were drawn after an overnight fast. Following this, all subjects underwent a standard OGTT, as previously described (17). In short, the OGTT was done according to standard procedures. Patients received a glucose solution concentrated 1.75 g glucose/kg body weight (maximum 75g glucose) and blood sampling was performed at time points -5, 5, 10, 15, 30, 60, 90, 180 min. Blood was sampled through a venous catheter.

Glucose, triacylglycerides (TG) and high-density lipoproteins (HDL) were analyzed according to local protocols. In Uppsala, glucose was analyzed using an Architect c8000 instrument (Abbott Diagnostics, Solna, Sweden) and by a Gluco-quant Glucose-Kit (Roche Diagnostics, Mannheim, Germany) in Salzburg. Uppsala quantified TG and HDL using an Architect c800 instrument (Abbott Diagnostics) and in Salzburg an enzymatic photometric test (Modular Analytics System). Additional evaluation of LDL cholesterol was required, which was done with an enzymatic photometric test using Integra Manual by Roche Diagnostics. An enzyme-linked immunosorbent assay (ELISA) (Modular Analytics System, E-Modul by Roche Diagnostics) was used to analyze leptin and adiponectin. HbA1c was measured by reversed-phase chromatography (RP-HPLC). P-Modul, 917; Roche Diagnostics) was used. Validation of analyses was performed between the laboratories in Uppsala and Salzburg using reference blood samples.

Selected samples underwent immediate centrifugation at 2500g for 10 minutes at 4°C, subsequently aliquoted, and frozen at −80°C. Plasma was later used for central analyses of insulin and glucagon in Uppsala for both study centers. Single-plex enzyme-linked immunosorbent assay kits for each analyte were used (Mercodia AB, Uppsala, Sweden). Standardized control samples (Mercodia AB) were used to control for interplate variability.

### Assessment of insulin resistance

The following indices were used for the determination of insulin resistance (IR) and insulin sensitivity.

To measure hepatic insulin resistance the homeostatic model assessment (HOMA) (Wallace 2004) and the Hepatic Insulin Resistance Index (HIRI) were used. The HOMA- insulin resistance (IR) was calculated as the product of fasting glucose (mmol/L) and fasting insulin (µU/ml) divided by constant 22.5 (17, 18) and the HIRI measured as the product of the area under the curves (AUCs) of glucose and insulin for the first 30 min of the OGTT (19). Insulin sensitivity was calculated by the Single Point Insulin Sensitivity Estimator (SPISE), the newest biomarker for insulin sensitivity developed by Paulmichl et al (20) and the Matsuda Whole Body Insulin Sensitivity Index (WBISI). The SPISE is calculated by the product of the constant 600 and HDL-cholesterol^0,185^ divided by the product of triglycerides^0,2^ and BMI^1,33835^ and the WBISI (21, 22):


$$\frac{10,000}{\sqrt{\frac{\left(\text{fasting glucose }\times \text{ fasting insulin}\right)}{\text{mean glucose }\times \text{ mean insulin}}}}$$

### Statistical analysis

The data was analyzed descriptively showing results with mean and standard deviation for metric variables and number and percentages for categorical variables for a matched cohort.

NAFLD groups were matched 1:1 according to nearest neighbor algorithm for BMI-SDS. Matching performance was assessed applying Wilcoxon rank sum tests for unpaired samples pre- and post-matching resulting in significant differences before (p < 0.001) and non-significant results after matching (p = 0.383). Cohort size was reduced from n = 184 (79 vs. 105) to n = 132 (66 vs. 66) due to matching process.

Due to non-normality of the data, groupwise differences in glucagon were examined applying non-parametric Wilcoxon rank sum tests for unpaired samples. Further investigations of dependencies were assessed using univariate regression models. Our standardized multivariate model resulted, including significantly correlating parameters to fasting glucagon, from univariate models. In a second step the multivariate model size was reduced by exclusion of parameters which were not significantly different in the group differences. In a final step, variables were excluded for multicollinearity based on the variance inflation factor (VIF). The threshold for this exclusion was set to 10 as suggested in the literature (23).

All results are presented along with 95%-Confidence Intervals. Tests are performed at a significance level of 5%. P-values in multivariable models are corrected with Bonferroni-Holm method for multiple testing. No p-value correction for the remaining results. Statistical analysis was done with R (version 4.0.2). Important R-packages: leaps (selection algorithm), MatchIt (matching).

## Results

### Baseline characteristics

Clinical and anthropometric features of the study population are shown in **Table 1**. After matching for BMI-SDS the study population included 132 adolescents with overweight and obesity, mean BMI being 31.53 ± 6.86 kg/m2 and mean age 14.09 ± 2.34. Mean BMI between the NAFLD and non-NAFLD groups was similar, however the waist-to-hip ratio showed a significant difference between the groups (NAFLD 0.98 ± 0.08 vs. 0.94 ± 0.08, p = 0.02).

**Table 1:** Descriptive data of all patient (study population, N = 132) and comparison of baseline characteristic difference between NAFLD (n = 66) and non- NAFLD (n = 66) patients.

	Study population (N = 132)	NAFLD (n = 66)	non-NAFLD (n = 66)	p-value
**CLINICAL FEATURES**				
**Age (Years)**	14.09 ± 2.34	14.38 ± 2.33	14.03 ± 2.40	0.49
Tanner stage*	I: 10 (7.6%) II-IV: 83 (62.9%) V: 30 (22.7%) n.a.: 9 (6.8%)	I: 7 (10.1%) II-IV: 42 (63.6%) V: 13 (19.6%) n.a.: 4 (6.1%)	I: 3 (4.5%) II-IV: 41 (62.1%) V: 173(25.6%) n.a.: 5 (7.8%)	0.34
**BMI (mg/m2)#**	31.53 ± 6.86	32.28 ± 4.81	32.05 ± 4.96	0.74
**BMI-SDS#**	2.57 ± 1.12	2.84 ± 0.51	2.77 ± 0.49	0.38
**SBMI (kg/m2)**	33.98 ± 5.83	34.93 ± 3.52	34.63 ± 3.51	0.57
**Waist circumference (cm)#1**	101.95 ± 17.45	105.35 ± 13.25	103.85 ± 12.65	0.77
**Hip circumference (cm)**	107.81 ± 14.89	107.88 ± 11.57	110.45 ± 12.76	0.16
**Waist-to-hip-ratio**	0.94 ± 0.09	0.98 ± 0.08	0.94 ± 0.08	0.02
**RR systolic (mmHg)#3**	120.53 ± 11.57	121.51 ± 12.18	121.28 ± 10.55	0.99
**BODY FAT COMPOSITION**				
**Total body fat (%)**	42.50 ± 5.87	41.70 ± 6.42	41.54 ± 4.89	0.87
**MRI VAT volume (cm3)#2**	1463.46 ± 703.24	1753.07 ± 662.31	1302.27 ± 433.52	0.00
**MRI SAT volume (cm3)#2**	6332.09 ± 2789.08	6687.25 ± 2060.33	6625.70 ± 2127.87	0.99
**MRI liver fat content (%)#**	9.38 ± 10.26	15.58 ± 10.95	3.17 ± 0.97	0.00
**GLUCOSE METABOLISM**				
**Fasting glucose (mmol/L)**	5.15 ± 1.27	5.25 ± 2.01	5.01 ± 0.60	0.54
**OGTT 120 min. glucose (mmol/L)#**	6.72 ± 2.10	7.16 ± 2.99	6.41 ± 1.39	0.22
**Fasting insulin (pmol/L)#5**	111.85 ± 66.79	129.46 ± 66.40	104.00 ± 46.58	0.08
**HbA1c (mmol/mol)**	35.81 ± 7.08	37.47 ± 11.85	34.77 ± 2.33	0.15
**Fasting glucagon (pmol/L)**	11.94 ± 6.69	14.20 ± 8.54	10.36 ± 3.99	0.01
**METABOLIC INDICES**				
**SPISE#1**	5.47 ± 2.43	4.80 ± 1.24	5.12 ± 1.34	0.22
**WBISI#7**	5.47 ± 4.33	4.14 ± 2.60	4.89 ± 2.25	0.10
**HIRI#8**	47861.88 ± 30367.22	52950.30 ± 26984.56	45451.40 ± 21248.58	0.46
**HOMA-IR#6**	3.75 ± 2.51	4.41 ± 2.62	3.32 ± 1.54	0.06
**LIPID PROFILE**				
**Total cholesterol (mmol/L)**	4.12 ± 0.76	4.30 ± 0.87	4.13 ± 0.66	0.24
**LDL cholesterol (mmol/L)**	2.40 ± 0.69	2.58 ± 0.79	2.40 ± 0.61	0.08
**HDL cholesterol (mmol/L)#1**	1.25 ± 0.32	1.19 ± 0.24	1.28 ± 0.37	0.32
**Triglyceride (mmol/L)#**	1.17 ± 0.69	1.37 ± 0.73	1.06 ± 0.51	0.01
**LIVER FUNCTION**				
**AST (μkat/L)#4**	0.53 ± 0.31	0.64 ± 0.44	0.45 ± 0.25	0.00
**ALT (μkat/L)#**	0.55 ± 0.54	0.80 ± 0.79	0.37 ± 0.20	0.00
**GGT (μkat/L)#**	0.37 ± 0.28	0.48 ± 0.42	0.29 ± 0.11	0.00

Data are expressed a mean ± standard deviation (SD).

**p < 0.05.**

n = 104 for BMI, BMI-SDS, liver fat content, OGTT 120 min. glucose, triglycerides, ALT, GGT; #1n = 103 for waist circumference, HDL-cholesterol, SPISE; #2n= 102 for VAT and SAT volume; #3n = 101 for systolic blood pressure; #4n = 98 for AST; #5n = 75 for fasting insulin; #6n = 73 for HOMA-IR; #7n = 63 for WBISI; #8n = 60 for HIRI.

NAFLD, non-alcoholic fatty liver disease; n.a., not available; BMI, body mass index; BMI-SDS, body mass index standard deviation score; SBMI, smart BMI; RR, blood pressure; HbA1c, hemoglobin A1c; LDL, low density lipoprotein; HDL, high density lipoprotein; AST, aspartate aminotransferase; ALT, alanine aminotransferase; GGT, gamma glutamyl transferase; OGTT, oral glucose tolerance test; MRI, magnetic resonance imaging; VAT, visceral adipose tissue; SAT, subcutaneous adipose tissue; DSAT, deep subcutaneous adipose tissue; SSAT, superficial subcutaneous adipose tissue; SPISE, single point insulin sensitivity estimator; WBISI, whole-body insulin sensitivity index; HOMA-IR, homeostatic model assessment for insulin resistance; HIRI, hepatic insulin resistance index.

*Tanner staging I–V: I, prepubertal; II–IV, pubertal; V = post-pubertal.

Body fat composition variables presented a significant difference between the mean liver fat content (NAFLD 15.58 ± 10.95% vs. non-NAFLD 3.17 ± 0.97%, p=0.00) and the visceral adipose tissue (VAT) (NAFLD 1753.07 ± 662.31 vs. non-NALFD 1302.27 ± 433.52, p=0.00) in the matched study population (N=132). Of the biochemical parameters of glucose metabolism fasting insulin (NAFLD 129.46 ± 66.40 vs. non NAFLD 104.00 ± 46.58, p=0.08), fasting glucagon (NAFLD 14.20 ± 8.54 vs. non-NAFLD 10.36 ± 3.99, p=0.01) and HOMA-IR (NAFLD 4.41 ± 2.62 vs. non-NAFLD 3.32 ± 1.54, p=0.06) presented significant differences between the groups. Additionally, among the lipid profile markers significant differences between the groups could be observed between the LDL cholesterol (NAFLD 2.58 ± 0.79 vs. 2.40 ± 0.61, p=0.08) and the triglycerides (NAFLD 1.37 ± 0.73 vs. 1.06 ± 0.51, p=0.01). Finally, all enzymatic liver function parameters (ALT, AST, GGT) presented significant differences between the NAFLD and non NAFLD patients (AST: NAFLD 0.64 ± 0.44 vs. non NAFLD 0.45 ± 0.25, p=0.00, ALT: NAFLD 0.80 ± 0.79 vs. non-NAFLD 0.37 ± 0.20, p=0.00, GGT: NAFLD 0.48 ± 0.42 vs. non NAFLD 0.29 ± 0.11, p=0.00). HOMA-IR was the only metabolic index that showed significant differences between the two groups (NAFLD 4.41 ± 2.62 vs. non NAFLD 3.32 ± 1.54, p=0.06).

### Fasting glucagon concentrations correlate with metabolic parameters in adolescents with NAFLD and non NAFLD

**Figure 1:**
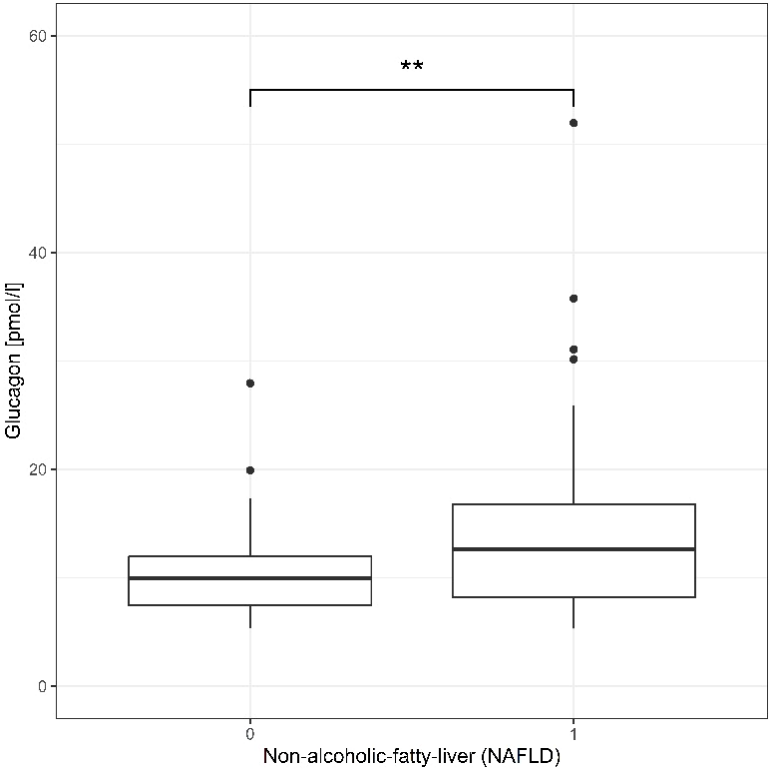
Fasting glucagon concentration pmol/L of participant without NAFLD (0, n = 66) and with NAFLD (1, n = 66) are shown a box plot in quartile and outliers. Wilcoxon test yielded a highly significant result p=0.0079 a **p < 0.01.

**Figure 1** demonstrates that fasting plasma glucagon levels are significantly different between the NAFLD and non-NAFLD group (p<0.01), N=132.

A univariate analysis for fasting plasma glucagon levels matched for BMI-SDS was constructed according to metabolic variables (glucose metabolism, liver function and lipid profile) such as body fat composition and clinical features. Variables of glucose metabolism (OGTT 120 min p=0.044, fasting insulin p<0.001, SPISE p=0.002, WBISI p=0.001, HIRI p<0.001, HOMA-IR p<0.001), of lipid profile (HDL cholesterol p=0.034, triglycerides p<0.001) and liver function (AST p=0.010, p=0.010, p=0.009), indicated a significant relationship with glucagon (**Table 2**).

**Table 2:** Univariate analysi for fasting glucagon (pmol/L) in patient with overweight/obesity (n = 104#) matched for BMI-SDS.

	coefficient	p-value	R2
**CLINICAL FEATURES**			
BMI (mg/m2)	0.25	0.073	0.022
BMI-SDS	3.50	0.010**	0.054
Waist circumference (cm)	0.09	0.083	0.020
RR systolic (mmHg)	0.05	0.359	-0.002
**BODY FAT COMPOSITION**			
MRI VAT volume (cm3)	0.01	<0.001***	0.194
MRI SAT volume (cm3)	0.00	0.341	-0.001
MRI liver fat content (%)	0.28	<0.001***	0.144
**GLUCOSE METABOLISM**			
OGTT 120 min. glucose (mmol/L)	0.54	0.044*	0.030
Fasting insulin (μIU/mL)	0.07	<0.001***	0.248
SPISE	-1.61	0.002**	0.079
WBISI	-1.28	0.001***	0.152
HIRI	0.00	<0.001***	0.234
HOMA-IR	1.72	<0.001***	0.211
**LIPID PROFILE**			
HDL cholesterol (mmol/L)	-4.51	0.034*	0.034
Triglyceride (mmol/L)	4.34	<0.001***	0.160
**LIVER FUNCTION**			
AST (μkat/L)	4.55	0.010**	0.057
ALT (μkat/L)	2.61	0.010**	0.055
GGT (μkat/L)	5.45	0.009**	0.056

*p < 0.05, **p < 0.01, ***p < 0.001.

n = 104 for BMI, BMI-SDS, liver fat content, OGTT 120 min. glucose, triglycerides, ALT, GGT; n = 103 for waist circumference, HDL-cholesterol, SPISE; n = 102 for VAT and SAT volume, hsCRP; n = 101 for systolic blood pressure; n = 98 for AST; n = 78 for Il-6, TNF alpha; n = 75 for fasting insulin; n = 73 for HOMA-IR; n = 63 for WBISI; n = 60 for HIRI.

BMI, body mas index; BMI-SDS, body mas index standard deviation score; SBMI, smart BMI; RR, blood pressure; HbA1c, hemoglobin A1c; LDL, low density lipoprotein; HDL, high density lipoprotein; AST, aspartate aminotransferase; ALT, alanine aminotransferase; GGT, gamma glutamyl transferase; OGTT, oral glucose tolerance test; MRI, magnetic resonance imaging; VAT, visceral adipose tissue; SAT, subcutaneou adipose tissue; DSAT, deep subcutaneou adipose tissue; SSAT, superficial subcutaneou adipose tissue; SPISE, single point insulin sensitivity estimator; WBISI, whole-body insulin sensitivity index; HOMA-IR, homeostatic model assessment for insulin resistance; HIRI, hepatic insulin resistance index.

**Figure 2:**
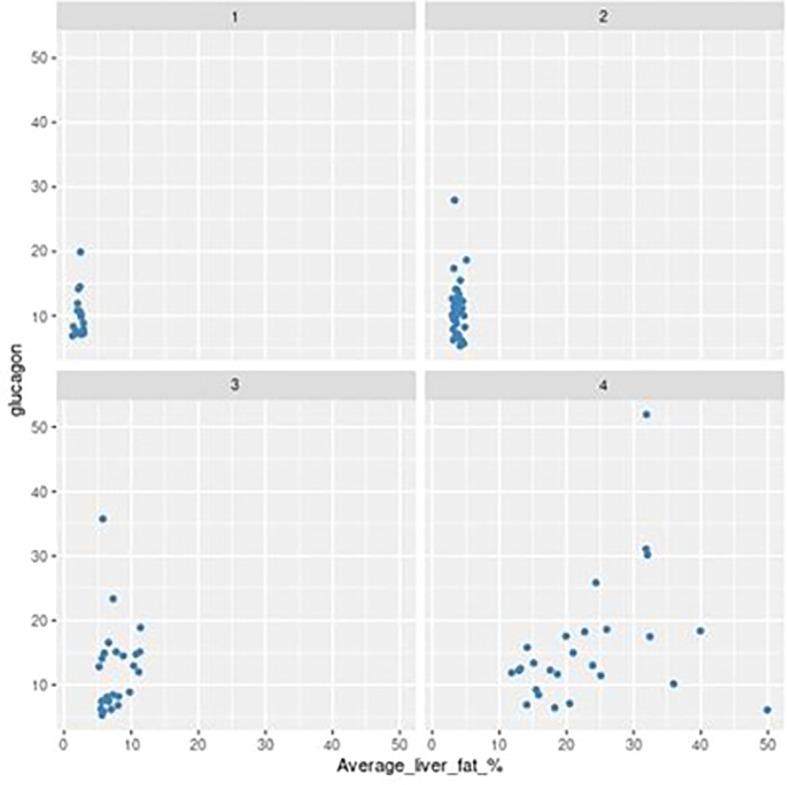
Scatter plot showing relationship between average liver fat (%) and fasting glucagon (pmol/L) grouped by MRI quartile (1-4).

**Figure 2** presents a scatterplot of all NAFLD patients stratified by liver fat content quartiles, resulting in the following cut-offs: 25% = 2.91% liver fat content, 50% = 5.11% liver fat content, 75% = 11.73% liver fat content. Looking at the relationship between glucagon and average liver fat within each quartile, a clear positive trend can be observed in quartile 4 (**Figure 2**).

### Predictors of fasting glucagon concentrations in patients with overweight and obesity

A standardized multivariate regression model was constructed based on the glucagon predictors BMI-SDS, average liver fat, fasting insulin, glucose at 120 min, SPISE and ALT in overweight and patients with overweight/obesity (**Table 3**). Significant drivers of this model (R2 = 0.336) were liver fat content (p=0.044), VAT (p=0.031), fasting insulin levels (p=0.016) and alanine aminotransferase (p=0.030). By contrast BMI-SDS (p=1.00), OGTT glucose 120 min (p=1.00) and SPISE (p=1.00) did not change the significant driving variables.

**Table 3:** Multivariate regression analysi with standardized coefficients: predictor of glucagon in a pediatric cohort with overweight/obesity (n = 73) matched for BMI-SDS (R2 = 0.336), correct by Bonferroni-Holm algorithm.

	Coefficient	p-value
**Anthropometric data**		
BMI-SDS	-0.10	1.000
MRI VAT volume (cm**3**)	0.45	0.031*
MRI liver fat content (%)	0.47	0.044*
**Metabolic data**		
OGTT 120 min. glucose (mmol/L)	-0.04	1.000
Fasting insulin (μIU/mL)	0.40	0.016*
SPISE	0.04	1.000
ALT (μkat/L)	-0.46	0.030*

^*^p < 0.05, tested for multicollinearity.

BMI-SDS, body mas index standard deviation score; MRI, magnetic resonance imaging; VAT, visceral adipose tissue; ALT, alanine aminotransferase; OGTT, oral glucose tolerance test; SPISE, single point insulin sensitivity estimator.

### Predictors of fasting glucagon concentrations in patients with NAFLD

A multivariate regression model to evaluate predictors of glucagon in NAFLD adolescents was applied, with average liver fat being a conditional variable in the models. The model (**Table 4**, R² = 0.509) included VAT and HIRI index. VAT (p=0.017) and the HIRI index (p=0.003) resulted with a predictive effect for hyperglucagonemia in pediatric NAFLD.

**Table 4:** Multivariate regression analysi with standardized coefficients: determinant of hyperglucagonemia in NAFLD (n = 66) matched for BMI-SDS (Model R² = 0.509).

Model	Coefficient	p-value
Average liver fat, %	0.097620	0.477
VAT, cm³	0.006884	0.017*
HIRI	0.000187	0.003*

*p < 0.05, tested for multicollinearity.

VAT, visceral adipose tissue; HIRI, Hepatic Insulin Resistance Index.

## Discussion

This is the first study to examine predictors of hyperglucagonemia in a pediatric NAFLD population. Our data identify visceral adipose tissue and the HIRI index as surrogate for hepatic insulin resistance as determinants of hyperglucagonemia in pediatric NAFLD.

Obesity significantly increases the risk of NAFLD, effecting the pediatric population with obesity in large numbers (24). VAT and WHR, but not BMI, predicted increased levels of glucagon in our cohort. This implies that the accumulation of visceral fat rather than adiposity associates with deranged glucagon metabolism. This is in accordance with the findings of Manell et al., who concluded that high levels of glucagon are related to VAT, rather than liver fat content, pancreas fat content and subcutaneous adipose tissue (SAT) (3). A positive relation between fasting hyperglucagonemia and increased WHR could also be observed in an adult cohort where WHR turned out to be the best anthropometric predictor of NAFLD (25, 26). This indicates that VAT and central adiposity are closely related to NAFLD occurrence (27). Analyzing data from a different pediatric cohort, our group previously showed that increased WHR is related to increased VAT and fasting insulin levels in children with obesity and increased hepatic liver fat content (28). This is in keeping with a plethora of studies reporting that WHR and VAT can be identified as indirect parameters of insulin resistance (29, 30).

In our study, univariate analysis not only indicated a significant relationship of fasting insulin, VAT, MRI liver fat content with glucagon in our cohort of children and adolescents with overweight and obesity, but also with the liver enzyme alanine transaminase (ALT). Alanine transaminase levels are accepted as surrogates of NAFLD in clinical practice (30, 31), although liver enzymes are known to be limited in sensitivity and specificity in the diagnosis of pediatric NAFLD (31, 32). It is worth mentioning in this context, that children with overweight/obesity and elevated ALT values had a more than 2-fold increased risk for future dysglycemia independent of age, sex and BMI-SDS in a survival analysis of up to 11 years of follow-up of 510 children with overweight and obesity from the Leipzig Childhood Cohort. Hence, elevated transaminases were suggested as an early predictor for glycemic deterioration (31).

The multiple regression model identified VAT and the HIRI index as the best predictive variables for hyperglucagonemia in our pediatric NAFLD cohort. The HIRI index is a dynamic surrogate index derived from the OGTT. Recent studies have evaluated the predictive accuracy of surrogate indices for hepatic insulin resistance derived from dynamic tests, such as the HIRI, suggesting that these are suitable alternatives to describe ß-cell function (20, 22, 33). Similar to our pediatric cohort, D’Adamo and Deivanayagam concluded that an increase in intrahepatic fat is associated with an increase in the HIRI index (34, 35). Additionally, when considering surrogates of insulin sensitivity (HOMA IR, HIRI), adult studies showed that increased circulating levels of fasting glucagon, together with increased insulin levels, are tightly coupled to a reduction of insulin sensitivity in individuals with normal and disturbed glucose metabolism (36–38). In our study cohort a similar relationship could be observed in children with NAFLD. This is an interesting aspect as direct glucagon suppression is caused by insulin stimulation as seen in non-diabetic subjects (39). Recently, evidence of a liver-alpha cell axis in humans was introduced (40, 41). The concept claims that fat accumulation in the liver attenuates the sensitivity of hepatocytes towards glucagon causing impaired hepatic glucagon signaling and consequently results in hyperglucagonemia (41, 42). Our data are in line with previous adult studies (41–43) supporting the existence of such a liver-alpha cell feedback loop as early as during childhood. Faerch et al. showed that glucose regulation during development of insulin resistance was linked not merely with hypersecretion of insulin, but also with a reduced capability to acutely suppress glucagon after glucose intake in adults (37). Hypersecretion of glucagon from pancreatic alpha cells has hence been suggested to be due to an impairment of hepatic glucagon signaling, which then, due to decreased glucagon-induced amino acid turnover, would result in hyperaminoacedemia (14). Lischka et al. recently reported higher levels of plasma branched-chain amino acids in children with NAFLD, suggesting that BCAAs could be an important link between obesity and other metabolic pathways (43). However, the association between amino acid and glucagon metabolism in pediatric NAFLD has yet to be studied.

There are some strengths and limitations that need to be acknowledged. The primary strength of the study was a relatively large study cohort of children with MRI diagnosed NAFLD. Although the gold standard of diagnosis of different stages of NAFLD – steatosis to fibrosis/cirrhosis - would be liver biopsy, MRI scans are well suited to quantify liver fat content which was the aim of this study (16). A limitation of the current study is that the conclusions cannot be translated to other ethnic groups other than Caucasian. Further, a more detailed characterization according to pubertal stages matched by age and BMI-SDS was not feasible due to statistical limitations related to sample size.

In summary, our results identify that average liver fat content is predictive in pediatric overweight and obesity. Visceral adipose tissue (VAT) and the HIRI index were identified as determinants of hyperglucagonemia in pediatric NAFLD, but not average liver fat content.

## Data availability statement

The raw data supporting the conclusions of this article will be made available by the authors, without undue reservation.

## Ethics statement

The studies involving human participants were reviewed and approved by ethical committee of city of Salzburg. Written informed consent to participate in this study was provided by the participants’ legal guardian/next of kin.

## Author contributions

KMa and DW conceived the idea and designed the study. KR designed the tables. SS performed the statistical analysis. HarM, PB, AF, HanM, TP, HA, JK and KMö revised the paper. KMa and DW wrote the manuscript draft. All authors revised and accepted the final version of the manuscript.

## Conflict of interest

The authors declare that the research was conducted in the absence of any commercial or financial relationships that could be construed as a potential conflict of interest.

## Publisher’s note

All claims expressed in this article are solely those of the authors and do not necessarily represent those of their affiliated organizations, or those of the publisher, the editors and the reviewers. Any product that may be evaluated in this article, or claim that may be made by its manufacturer, is not guaranteed or endorsed by the publisher.
